# From many voices, one question: Community co-design of a population-based qualitative cancer research study

**DOI:** 10.1371/journal.pone.0309361

**Published:** 2024-08-26

**Authors:** Susannah K. Ayre, Elizabeth A. Johnston, Xanthia E. Bourdaniotis, Leah Zajdlewicz, Vanessa L. Beesley, Jason D. Pole, Aaron Hansen, Harry Gasper, Danica Cossio, Gemma Lock, Belinda C. Goodwin

**Affiliations:** 1 Cancer Council Queensland, Brisbane, Queensland, Australia; 2 School of Exercise and Nutrition Sciences, Queensland University of Technology, Brisbane, Queensland, Australia; 3 Population Health Program, QIMR Berghofer Medical Research Institute, Brisbane, Queensland, Australia; 4 Psychedelic Medicine and Supportive Care Lab, QIMR Berghofer Medical Research Institute, Brisbane, Queensland, Australia; 5 School of Psychology, The University of Queensland, Brisbane, Queensland, Australia; 6 School of Nursing, Queensland University of Technology, Brisbane, Queensland, Australia; 7 Centre for Health Services Research, The University of Queensland, Brisbane, Queensland, Australia; 8 Dalla Lana School of Public Health, The University of Toronto, Toronto, Ontario, Canada; 9 Icon Cancer Centre, Brisbane, Queensland, Australia; 10 Princess Alexandra Hospital, Brisbane, Queensland, Australia; 11 School of Medicine, University of Queensland, Brisbane, Australia; 12 Toowoomba Base Hospital, Toowoomba, Queensland, Australia; 13 Cancer Alliance Queensland, Brisbane, Queensland, Australia; 14 Centre for Health Research, University of Southern Queensland, Springfield, Queensland, Australia; 15 School of Population and Global Health, University of Melbourne, Melbourne, Victoria, Australia; North South University, BANGLADESH

## Abstract

**Purpose:**

This study formed the development stage of a population-based survey aiming to: (i) understand the needs and experiences of people affected by cancer in Queensland, Australia and (ii) recruit a pool of participants for ongoing cancer survivorship research. The current study aimed to co-design and test a single qualitative survey question and study invitation materials to maximise acceptability of, and participation in, the survey and future research.

**Methods:**

Fifty-two community members, including cancer survivors and caregivers, participated across 15 co-design workshops and 20 pretest interviews. During workshops, participants generated and refined ideas for an open-ended survey question and provided feedback on a study invitation letter. The use of a single, open-ended question aims to minimise participant burden while collecting rich information about needs and experiences. The research team then shortlisted the question ideas and revised study invitation materials based on workshop feedback. Next, using interviews, community members were asked to respond to a shortlisted question to test its interpretability and relevance and to review revised invitation materials. Content analysis of participant feedback was used to identify principles for designing study materials.

**Results:**

Principles for designing qualitative survey questions were identified from participant feedback, including *define the question timeframe and scope; provide reassurance that responses are valid and valued;* and *use simple wording*. Principles for designing study invitation materials were also identified, including *communicate empathy and sensitivity; facilitate reciprocal benefit;* and *include a ‘human element’*. The qualitative survey question and study invitation materials created using these principles were considered relevant and acceptable for use in a population-based survey.

**Conclusions:**

Through community consultation and co-design, this study identified principles for designing qualitative data collection and invitation materials for use in cancer survivorship research. These principles can be applied by other researchers to develop study materials that are sensitive to the needs and preferences of community members.

## Introduction

In recent decades, an ageing population has resulted in more people being diagnosed with cancer, and improvements in cancer detection and treatment means that people are living longer post-diagnosis [1, 2]. While people diagnosed with cancer have unique disease trajectories, a common experience of cancer and its treatment is the widespread impact on a person’s health and wellbeing, often continuing well after treatment completion [3]. Informal caregivers (i.e., family and friends) are closely involved in supporting their loved ones to manage the impact of cancer and its treatment, often with minimal preparation for their caregiving role [4]. Consequently, caregivers can experience poor health and wellbeing due to the prolonged stress and physical demands involved [5]. In the context of increasingly resource-constrained healthcare systems, there is a growing need for community-based supportive care services that are effective in supporting the increasing number of cancer survivors and caregivers [6].

The delivery of effective supportive care services cannot be achieved without a comprehensive understanding of the needs and experiences of the cancer survivors and caregivers for whom these services are provided to. Evidence indicates that patient- and family-centred interventions result in higher satisfaction with healthcare, increased knowledge and skills for managing self-care behaviours, reduced reliance on healthcare services, and improved quality of life for both patients and caregivers [7]. Many instruments have been used in research and clinical practice to assess needs [8, 9]. The Supportive Care Needs Survey-Short Form (SCNS-SF34) [10], Cancer Survivors’ Unmet Needs (CaSUN) [11], Comprehensive Needs Assessment Tool for Cancer Caregivers (CNAT-C) [12], and Supportive Care Needs Survey for partners and caregivers (SCNS-P&C) [13] are among the most widely used. However, such instruments rely on set items with pre-determined responses that may not capture the full scope of needs and do not allow respondents to express their experiences in their own words [14]. Thus, qualitative survey methods are necessary to achieve a comprehensive understanding of the needs and experiences of people affected by cancer at the population level. To date, no suitable qualitative survey exists, providing an opportunity for community participation in co-designing materials to capture the supportive care needs and experiences of people affected by cancer.

Co-design research methods encompass various levels of consumer engagement in the research process, including participation in study conceptualisation, design, conduct, and reporting [15]. Consumer involvement in research can contribute to better study outcomes, including higher enrolment and retention rates [16]. Additionally, thorough testing of study materials with consumers is recommended to ensure materials are easy to understand, sensitively worded, and able to elicit meaningful data to address research aims [17].

Through active and repeated engagement with community members, this study aimed to: (i) develop and test a qualitative survey question for collecting rich information about the supportive care needs and experiences of people affected by cancer, and (ii) design study invitation materials that are relevant and acceptable to cancer survivors and their caregivers. The use of a single, open-ended question aims to minimise participant burden and maintain a broad investigation of supportive care needs, rather than the traditional itemised approach. The materials developed in this study will be used in a new population-based study for understanding the needs and experiences of adults affected by cancer in Queensland, Australia and establishing a research-ready pool of participants to take part in ongoing cancer survivorship research. Findings from the current study will provide principles for researchers to apply when designing qualitative data collection tools and study invitation materials for research into supportive care needs and experiences, particularly in the cancer context.

## Materials and methods

**Fig 1:**
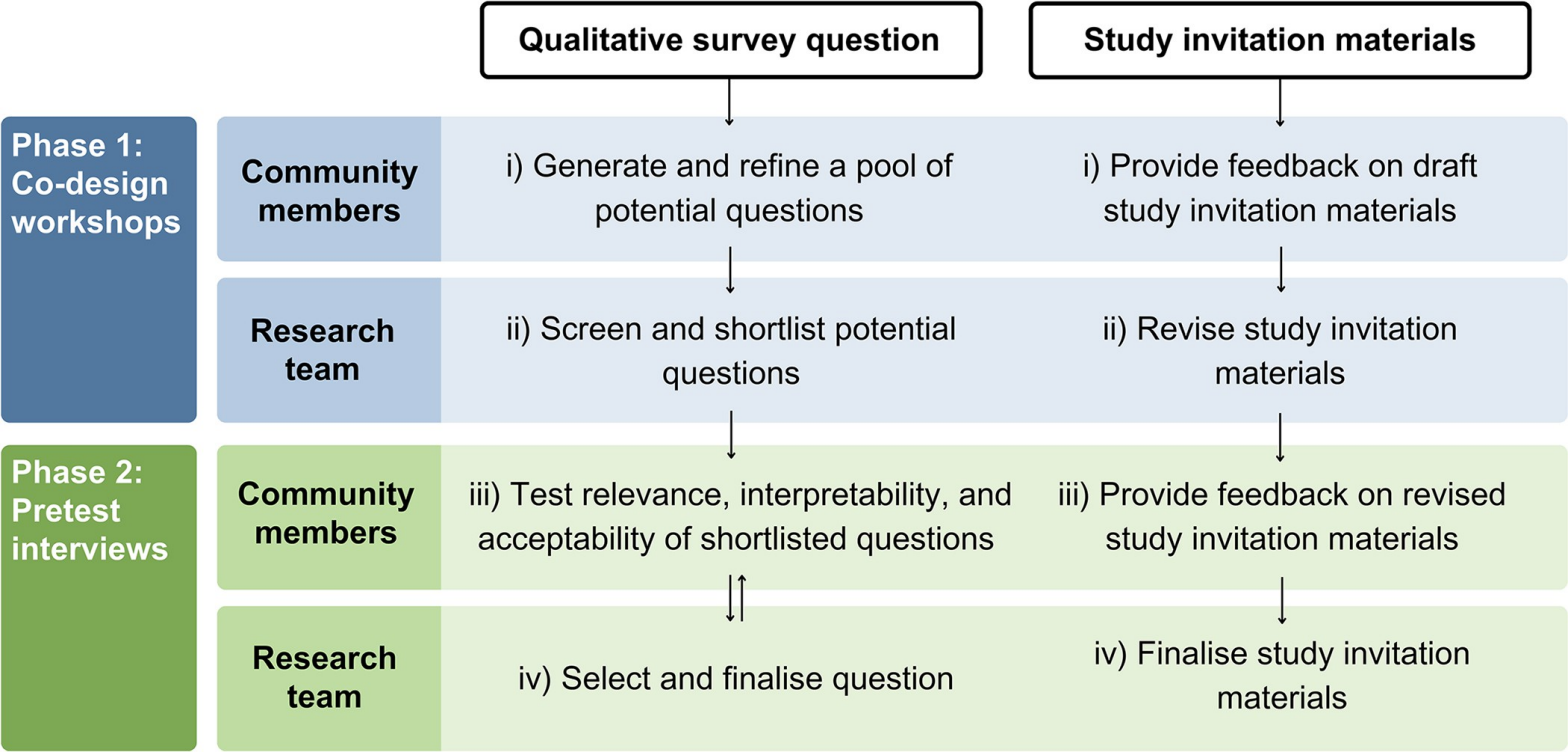
Overview of study procedures to co-design materials for a population-wide study on the supportive care needs and experiences of people affected by cancer.

This study comprised two phases of qualitative research: 1) *co-design workshops* and 2) *interviews*. As outlined in **Fig 1**, both phases included community members and the research team working collaboratively to co-design a qualitative survey question and study invitation materials. Ethical approval for this study was obtained from the University of Southern Queensland Human Research Ethics Committee (ref: ETH2023-0140). This study is reported as per the Consolidated Criteria for Reporting Qualitative Research (COREQ) Checklist [18] (see **S1 Table**).

### Participants and recruitment

This study was conducted in Queensland, Australia. Queensland has almost one third of its population living outside of major cities [19], which presents several challenges to accessing cancer treatment and support due to the greater travel distances and costs involved [20]. For both the co-design workshops and interviews, eligible participants were aged 18 years or older and English-speaking. Additional eligibility criteria for interview participants included a personal experience with cancer, either as a survivor or caregiver. Digital and printed recruitment flyers were distributed via networks associated with Cancer Council Queensland or the broader research team between 11 October 2023 and 14 February 2024. To support the recruitment of priority populations, such as culturally and/or linguistically diverse (CALD) groups, the research team submitted study information to a health consumers network [21] for inclusion in their e-newsletter. As data collection advanced, recruitment was supplemented through snowball sampling, with workshop participants invited to share recruitment flyers with friends, family members, and colleagues.

Prospective participants self-enrolled into a workshop and/or interview via an online participant information and consent form administered through REDCap (hosted by the University of Southern Queensland) [22, 23]. Consenting participants were then contacted by telephone and/or email to arrange a time for their participation. For the interviews, new participants were recruited alongside eligible workshop participants to obtain feedback from people with and without prior knowledge of the study.

Recruitment for the co-design workshops and interviews continued until a diverse sample had been achieved and the research questions had been adequately explored, determined by the authors through concurrent data collection and analysis. Due to the large number of online registrations for the interviews, new and existing participants were purposively sampled based on their demographic characteristics, including gender, ethnicity, Indigenous status, and geographical location, as well as their experience with cancer (i.e., survivor or caregiver) to ensure that diverse perspectives were represented [24]. To acknowledge their contributions to the study, workshop and interview participants received a voucher valued at AU$100.00 (approx. 120 minutes) and AU$50.00 (approx. 60 minutes), respectively.

### Data collection

#### Phase 1: Co-design workshops

Each workshop included two to four participants. As participants were grouped based on convenience, each workshop included a combination of cancer survivors, caregivers, and/or other community members. The workshops were facilitated by two female researchers with undergraduate or postgraduate degrees in health science fields and training in qualitative data collection (SA, XB, and/or EJ). The facilitators had no prior relationship with the participants. At the start of each workshop, the facilitators introduced themselves, including their role in the research team and their academic background. Workshops were conducted as either online (*n* = 9), in-person (*n* = 1), or hybrid (i.e., online and in-person) (*n* = 5) sessions using Microsoft Teams. In-person participants attended the session at one of two not-for-profit organisations, where participants were provided with the relevant materials (e.g., pen, paper). Participants attending online were asked to source these materials themselves. Workshops were audio-recorded and transcribed via Microsoft Teams. The workshops comprised several activities, with accompanying information and instructions provided on presentation slides. An overview of the workshop protocol is available in **S2 Table**.

The first activity in the workshop used the nominal group technique [25] to generate and refine a pool of qualitative questions that could be used in the population-based survey for understanding the supportive care needs and experiences of people affected by cancer. This technique fosters balanced participation, serving as an effective and efficient method for achieving group consensus [25]. First, participants were asked to individually brainstorm ideas for how to word the qualitative question. A preamble to the question drafted by the research team was shared with participants as a prompt for question generation (see **S2 Table** for further details). Participants were instructed that this question should be a standalone, broad, open-ended question that allows respondents to describe their needs and experiences using their own words, with the purpose of generating data that would inform service delivery at Cancer Council Queensland. Second, participants shared their questions with the group following a ‘round-robin’ process, during which a facilitator typed the questions verbatim onto the presentation slides. Third, participants collectively reflected on the proposed questions, sought clarification from one another, and adjusted wording as needed (e.g., removing potentially insensitive words). Finally, participants privately voted on their two most preferred questions using an online or paper-based poll. Votes were tallied to identify questions with the highest scores which were then presented to the group for further discussion and refinement, resulting in a total of one to five questions per group.

The second activity in the workshop involved participants providing feedback on the wording and format of an invitation letter for the population-based survey. This letter was drafted by the research team based on an example from a previous registry study. It comprised a single page of information about the research project, including its aims, instructions on how to participate, and permissions to recruit participants via the registry. During the workshops, participants were also prompted to discuss an appropriate time post-diagnosis to invite individuals to complete the survey.

Key principles endorsed by workshop participants for developing a qualitative survey question were presented to the investigator team, alongside questions that were open-ended, broad in scope, and aligned with these principles (see *Data analysis* section for methods used to identify key principles). The investigator team, comprising clinicians, researchers, and academics with expertise in cancer survivorship, supportive care, medical oncology, behavioural science, and digital health (*n* = 7; see **S3 Table**), were asked to rank their five most preferred questions (1 = ‘most preferred’, 5 = ‘least preferred’) via an anonymous online survey in REDCap [22, 23]. Four of the highest ranked questions were shortlisted for testing during interviews. Study invitation materials were also revised based on key principles endorsed by participants in the workshops.

#### Phase 2: Interviews

Online semi-structured interviews were conducted, audio-recorded, and transcribed using Microsoft Teams. Each interview was facilitated by one researcher (SA or XB). The interviews served as an opportunity for member checking of findings from the workshops. A summary of the interview protocol is available in **S4 Table**. Participants firstly reviewed the revised invitation materials and provided feedback on their readability and design. They were also asked to discuss an appropriate time for sending individuals a reminder letter to complete the survey. Participants were then randomly presented with one of the four shortlisted questions and given five uninterrupted minutes to respond to and submit their written response via the online chat function. Drawing on principles of the ‘think aloud’ method [26], participants then verbalised their thoughts, assumptions, and decisions while reading, interpreting, and responding to the question. Scripted and spontaneous probes were used to clarify interpretation as needed. Participants were then presented with the three alternative questions and asked to nominate their preferred question based on interpretability and relevance. After 14 interviews, there was a clear consensus on the most appropriate question to include in the survey (*n* = 13 votes). An additional six interviews were then conducted, focusing solely on this question. The interviews used the ‘think aloud’ method [26] to finalise the wording of this question and confirm its interpretability and relevance. Following the 20 interviews, feedback on the revised study invitation materials was applied by the research team if considered feasible and relevant to the research aims.

#### Demographic survey

Age, gender, country of birth, language spoken at home, ethnicity, postcode of residential address, and personal history with cancer (including patient or caregiver status) were collected via an online survey in REDCap [22, 23] at the start of each co-design workshop. For participants who did not attend a workshop, demographic data were collected through structured questions at the end of their interview.

### Data analysis

Descriptive statistics were used to summarise participant characteristics for the co-design workshops and interviews. Transcripts generated via Microsoft Teams were reviewed for accuracy alongside the audio recordings. Transcripts were then analysed using content analysis to identify key principles endorsed by participants for developing a qualitative survey question and designing study invitation materials. Content analysis involves the systematic coding of text into categories based on the words and language used, centring participants’ voice in the analysis [27]. Each transcript was coded by one author (XB, SA, or EJ), with decisions regularly discussed with other authors and documented using an audit trail.

## Results

**Fig 2:**
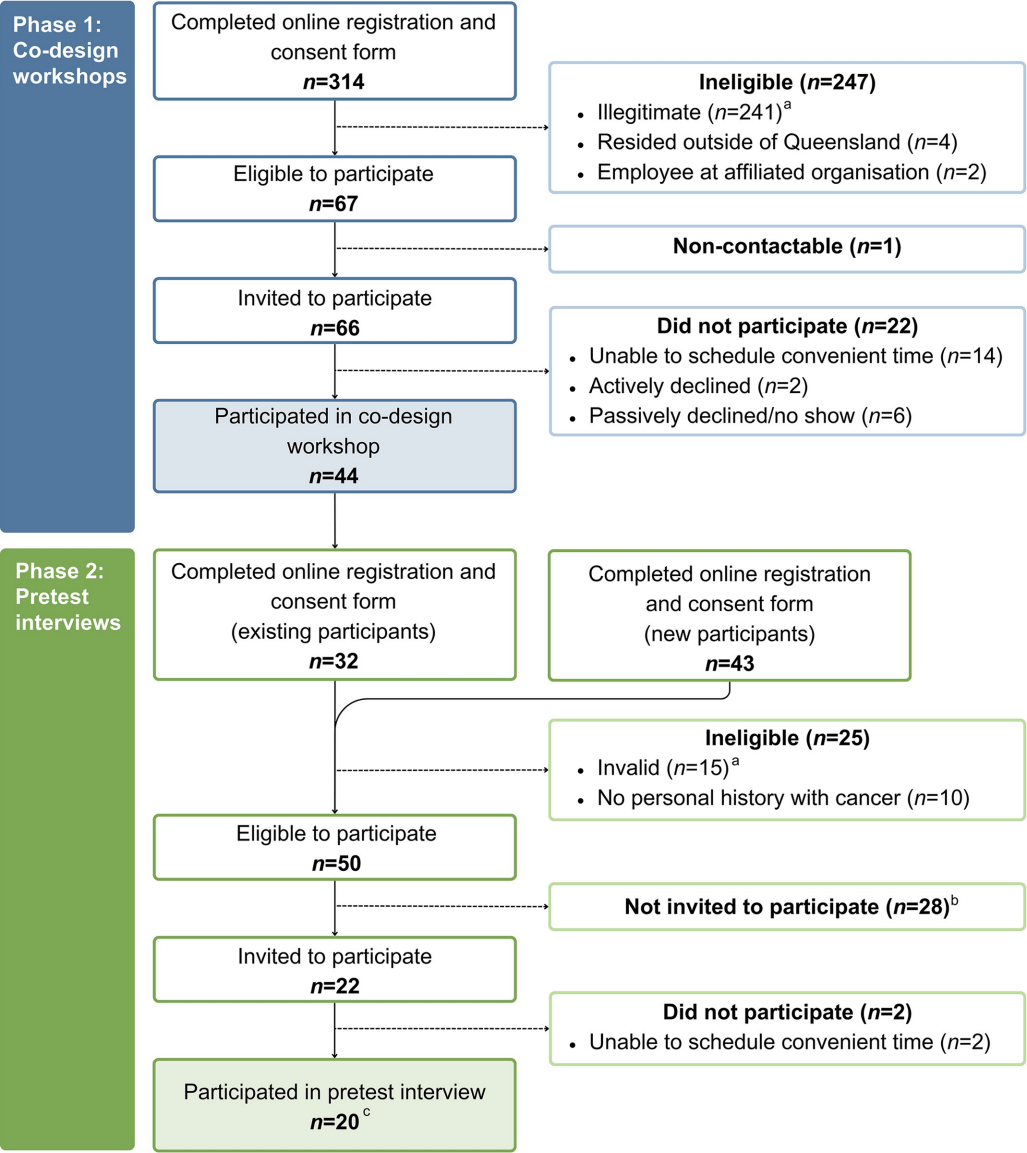
Flowchart of participant recruitment and selection for the co-design workshops and interviews to design materials for a population-wide study on the supportive care needs and experiences of people affected by cancer. ^a^Invalid responses were identified based on a combination of factors (e.g., duplicated IP addresses with different names, invalid postcodes or phone numbers, replicated responses in a short period of time, unusual completion times). Where necessary, responses flagged as potentially invalid were investigated further through phone and/or email contact. ^b^Individuals were purposively selected to achieve maximum variation in the age, gender, ethnicity, and geographic location of participants. ^c^12 of the 20 participants in the interviews also participated in a co-design workshop.

In total, 15 co-design workshops with 44 participants and 20 interviews were completed (see **Fig 2**). Twelve of the 20 interview participants had also completed a co-design workshop. The characteristics of participants in the two phases of consumer consultation are summarised in **Table 1**. Both phases included representation from population subgroups, including 5–7% who identified as Aboriginal and/or Torres Strait Islander, 9–15% who used English as a second language, 20% who were born overseas, and 27–30% who lived in a rural area.

**Table 1:** Characteristics of participants in the co-design workshops (*n* = 44) and interviews (*n* = 20) to design materials for a population-wide study on the supportive care needs and experiences of people affected by cancer.

Characteristic	Co-design workshops N (%)^a^	Interviews N (%)^a^
**Age (years)**		
** Median**	43	39
** Range**	23–79	30–64
**Gender**		
** Female**	32 (73%)	12 (60%)
** Male**	12 (27%)	8 (40%)
**Born overseas**		
** No**	33 (75%)	16 (80%)
** Yes**	9 (20%)	4 (20%)
** Unknown**	2 (5%)	-
**Ethnicity**		
** Caucasian**	36 (82%)	15 (75%)
** Asian**	5 (11%)	4 (20%)
** Aboriginal and/or Torres Strait Islander**	3 (7%)	1 (5%)
**Language spoken at home**		
** English only**	38 (86%)	17 (85%)
** Other**	4 (9%)	3 (15%)
** Unknown**	2 (5%)	-
**Geographic remoteness (ARIA)**		
** Major city**	32 (73%)	14 (70%)
** Regional or remote**	12 (27%)	6 (30%)
Area-level socioeconomic status (SEIFA)^b^		
** High**	24 (54%)	7 (35%)
** Medium**	13 (30%)	7 (35%
** Low**	7 (16%)	6 (30%)
**Personal experience with cancer**		
** Cancer survivor**	16 (36%)	10 (50%)
** Cancer caregiver**	17 (39%)	9 (45%)
** Both**	2 (5%)	1 (5%)
** Neither**	9 (20%)	-
**Time since diagnosis**		
** <1 year**	4 (9%)	2 (10%)
** 1–5 years**	6 (14%)	3 (15%)
** >5 years**	7 (16%)	5 (25%)
** Unknown**	1 (2%)	1 (5%)
** Not applicable**	26 (59%)	9 (45%)
**Duration of caregiving**		
** <1 year**	7 (16%)	3 (15%)
** 1–5 years**	6 (14%)	5 (25%)
** >5 years**	5 (11%)	1 (5%)
** Unknown**	1 (2%)	-
** Not applicable**	25 (57%)	10 (50%)

**Abbreviations:** ARIA, Accessibility/Remoteness Index of Australia; SEIFA, Socio-Economic Indexes for Areas

Number and percentage unless otherwise stated.

Higher scores indicate higher relative socioeconomic advantage and lower relative socioeconomic disadvantage in general (vice versa for lower scores). High = deciles 7–10, medium = deciles 4–6, low = deciles 1–3.

### Qualitative survey question

Analysis of the 15 co-design workshop responses yielded 16 eligible questions (see **Table 2**). Additionally, 10 principles were identified from participant feedback during the workshops for developing qualitative data collection tools (see **Table 3**). These principles were to: *avoid assumptions and leading questions; define the question timeframe and scope; directly address the respondent; foster a collectivist perspective; gather experiential data from respondents for researchers to identify solutions; prompt an open-minded response; provide reassurance that responses are valid and valued; use an engaging design and accessible formatting* (e.g., large and easy to read text)*; use sensitive language;* and *use simple wording*.

**Table 2:** Eligible questions generated in co-design workshops for designing a population-wide study on the supportive care needs and experiences of people affected by cancer.

Eligible questions^a^	
**1**	What is the most important thing for you right now?
**2**	**Please tell us how we can support you right now?**
**3**	**How has cancer affected your life and what do you need right now?**
**4**	**What support do you need right now based on your experience?**
**5**	**Please tell us about your experiences since diagnosis and what would help you most right now?**
**6**	Tell us what we need to know right now.
**7**	What would you find helpful right now?
**8**	What does your experience look like right now?
**9**	What support do you need right now?
**10**	How can we best support you to ensure your physical, emotional, and practical needs are met right now?
**11**	For you to feel supported, what do we need to do right now?
**12**	How can we support you right now?
**13**	How has your life been impacted right now?
**14**	Please share both your positive and negative experiences since diagnosis.
**15**	What kind of help do you need right now?
**16**	Please share your experiences and challenges since your diagnosis.

The order of questions was randomly assigned for coinvestigator voting and listed as shown in this table. Questions in bold type were the four highest ranked questions for further testing in interviews based on preferential voting by the co-investigator team.

**Table 3:** Principles endorsed by participants during co-design workshops for developing a qualitative survey to collect information on the supportive care needs and experiences of people affected by cancer in a population-based study.

Principle	Explanation	Illustrative quotes from co-design workshops^a^
**Avoid assumptions and leading questions**	Avoid making assumptions about how respondents might experience a cancer diagnosis, to ensure that those with diverse perspectives feel included, and to promote varied responses. For example, terms that imply a negative experience, such as ‘challenges’ and ‘struggles’, could limit the range of responses provided.	• **P1:** “Leaving it broader gives opportunity to include [responses like], ‘I did have a good support service, these people were really valuable’”. **(Workshop O)** • **P23:** “[If asking respondents what services are lacking], you’re assuming that people think they are lacking… There could be people that are thrilled with what they are receiving. . . Assuming they’re lacking may not be helpful”. **(Workshop G)**
**Define the question timeframe and scope**	Provide guidance on what type of response would be relevant to the research aims. For example, include a timeframe for the question (e.g., ‘since diagnosis’) and prompts to define its scope. These prompts should encourage detail and allow for variation in respondents’ answers (e.g., ‘Be as specific as possible’ and ‘You might like to consider your personal experiences with employment, transport, grief…’). Including question prompts helps to minimise burden and manage participant expectations of the research outcomes.	• **P15:** “I need some specificity [about the timeframe]… Are you talking about [my needs and experiences] through treatment or are you talking about now, or are you talking about after? That’s the specificity I would need to answer that question”. **(Workshop J)** • **P35:** “The only thing I would suggest is prompting [people to think about] different types of experiences–financial, social, emotional, relational. I think a lot of people think, ‘I’m having chemo’ and ‘I’m having surgery’ and ‘I’m feeling sick’, but there is also the isolation… not being with your friends and family. That ties into mental health and wellness. Then [there are the] financial and practical [impacts] too”. **(Workshop C)** • **P38:** “Come up with a list of what Cancer Council Queensland can do… So, the person that is confronting this thing [thinks], ‘Well alright, I can get financial counselling from you or help getting to the hospital’. …I think it would help to provide a bit of an overview of the general types of services you might be referring to [in this question]”. **(Workshop B)**
**Directly address the respondent**	Use second-person language to directly address and engage the respondent.	• **P26:** “I like the ‘you’ and ‘we’ because it is more direct. It engages you and you feel that you are being addressed”. **(Workshop F)**
**Foster a collectivist perspective**	Frame the question so that it encompasses the broader community, rather than the individual alone. Framing the question as such may encourage answers from respondents who prioritise the experiences of others before their own or may not feel comfortable expressing their own need for support.	• **P23:** “[The question] could be more generally about… how can we make what we do even better for *everybody*? Because culturally that is how a lot of people think… They tend to identify the general experience for everyone rather than the individual”. **(Workshop G)** • **P26:** “[If asked about what support I need], back then, I would have said nothing. I would have been like, ‘No, I’m doing this myself. I can handle it’. Whereas if the wording… was like, ‘How can we support people with cancer?’, I’d probably be more likely to answer it… It would still really be about my experience, but it would be looking out for other people instead of myself”. **(Workshop F)** • **P42:** “From my partner who was volunteering [in a wigs service], she mentioned that ladies would go there, but they really weren’t worried about having a wig. They were getting the wig to not scare their grandkids… Some things they do is purely for [their family]… So, [I like] ‘What would make life easier for you and those you care for?’” **(Workshop A)**
**Gather experiential data from respondents for researchers to identify solutions**	Invite respondents to share their personal experiences, rather than only focusing on their needs or asking them to provide a solution to address their needs. Participants felt that solutions for addressing supportive care needs are best devised with researcher input.	• **P15:** “I feel like it’s difficult for people not really involved in [research] to come up with their own thoughts on what services [they need]. They don’t know what services are out there… ‘What services do I want? Well, what’s something that even exists?’ …I feel like if you are not someone who is involved in this area, it might be hard for you to come up with these ideas on your own”. **(Workshop J)** • **P30:** “I know when I was jotting down [ideas for] the wording of the question, I started with asking people to share their experiences, rather than detailing what support services they want. [The latter] seems a bit direct and too specific”. **(Workshop E)** • **P35**: “You often don’t know what you need… because your brain is in survival mode to get through each day. I like the idea of sharing your experience and then the people… behind the research can, with a trained eye, can look at what are people struggling with? What has their experience been? What are the gaps that we can see?” **(Workshop C)**
**Prompt an open-minded response**	Encourage respondents to think beyond the scope of support that is currently available.	• **P26:** “If you put ‘hope’ or ‘wish’ [in the question], it might help [respondents] expand [their answer]. It’d have them [think], ‘Oh, I wish someone could…’”. **(Workshop F)** • **P42:** “I feel like ‘In a perfect world…’ helps people think about not just what’s on offer, but [what they would want] if they could have anything. They will actually share anything they want. Rather than saying, ‘Okay, what support do I have?’ [and] writing that down”. **(Workshop N)**
**Provide reassurance that responses are valid and valued**	Include general prompts to provide reassurance that all answers are valid and valued. For example, ‘There are no wrong answers,’ and ‘You can share as much or as little as you feel comfortable with’.	• **P29:** “If you want people to give a lot of detail, you need to be explicit about that. For example, ‘In as much detail as you feel alright to share…’. **(Workshop E)** • **P30:** “Put in the question that, ‘It is as much as you feel comfortable to share’. So, [respondents] don’t feel like their response won’t be included if they don’t provide a lot of detail”. **(Workshop E)**
**Use an engaging design and accessible formatting**	Use formatting that encourages respondents to answer. For example, use large font, include the survey on one page, and do not use a character or word limit in the online survey.	• **P2**: “I think [the survey] should be on one page. From my own experience, what you read in the first paragraph, or the first couple of paragraphs, is what sells you… You don’t want to read a whole lot of stuff”. **(Workshop O)** • **P5:** “If there’s older people with… vision impairments, is the text big enough? Make sure it’s accessible”. **(Workshop N)** • **P28**: “Sometimes when people get on a roll telling their story, they need more [space]… If you limit [their answer] to 1,000 characters, people will think they are running out of character space, [and think], ‘Let me go back to see what I can eliminate to get my story in”. **(Workshop E)**
**Use sensitive language**	Avoid potentially insensitive terms, such as ‘live well,’ ‘journey,’ and ‘fighting’ as these terms imply a degree of choice, control, or contentment in a cancer diagnosis.	• **P6:** “Get rid of ‘fighting’. A lot of [cancer patients] don’t like it… Sometimes people don’t have a fight. Sometimes they do and they feel survival guilt… It is one of those triggering words”. **(Workshop N)** • **P28:** “I don’t like the word ‘journey’. It implies that cancer is a holiday and nothing about cancer is a holiday”. **(Workshop F)** • **P37:** “[Remove] ‘live well’ [from the preamble], especially for people that might have received a terminal diagnosis. Unfortunately, they are not going to live well. It is a hard slog through that”. **(Workshop B)**
**Use simple wording**	Use simple and short sentences. Avoid phrases that could be misinterpreted by people for whom English is a second language.	• **P23:** “Sentences such as, ‘What does this [support] *look like* to you?’ could be too vague… People might think you are asking for a physical description [of what the services look like]. …If they are older, or [use English as a] second language, that sentence wouldn’t make sense”. **(Workshop G)** • **P26:** “[Removing the term ‘services’ makes the question] more digestible, and not as intimidating and overwhelming for someone with lower literacy. It’s the word ‘services’ that intellectualises the question”. **(Workshop F)** • **P42:** “Keep the question as simple as possible so that more people can understand the question and therefore answer”. **(Workshop A)**

Participants have been designated a code to maintain their anonymity.

**Fig 3:**
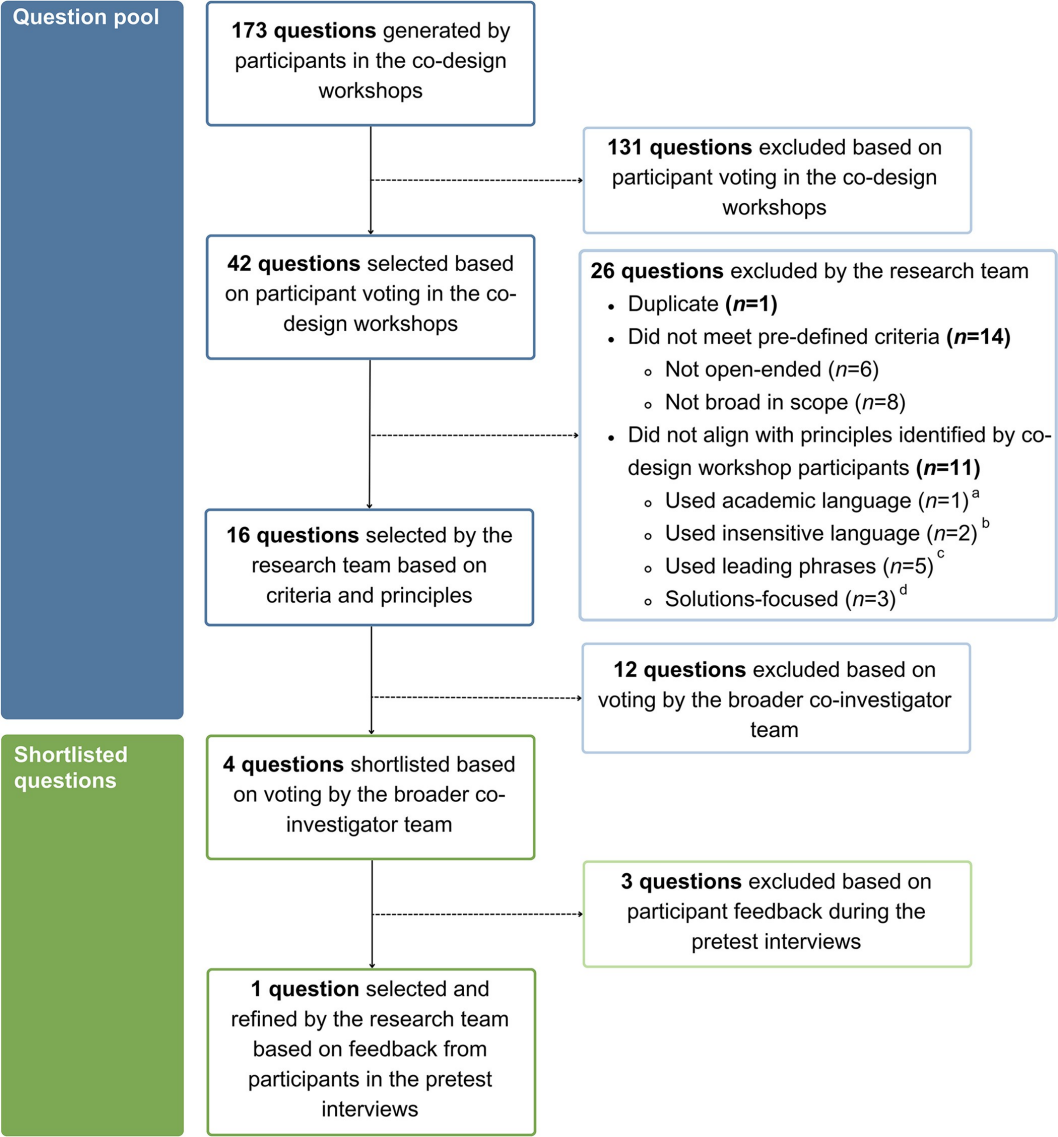
Flowchart of idea generation and shortlisting to develop a single, open-ended question for a population-wide study on the supportive care needs and experiences of people affected by cancer. ^a^i.e., Terms such as ‘quality of life’ and ‘daily living’. ^b^i.e., Terms such as ‘cancer journey’. ^c^i.e., Questions that imply a negative experience, such as ‘challenging’. ^d^i.e., Questions such as ‘What would make life easier for you?’.

A flowchart showing idea generation and shortlisting for the qualitative survey question is presented in **Fig 3**. During the workshops, participants generated a total of 173 questions with 42 questions selected through participant voting. Of these 42 questions, 14 (33%) did not meet pre-defined criteria (i.e., not open-ended, not broad in scope) and 1 question was a duplicate, warranting the exclusion of these questions. An additional 11 (26%) questions were excluded for not aligning with the principles endorsed by workshop participants. Although participants advocated for including a timeframe within the question, a limited number of questions aligned with this principle. Rather than excluding these questions, a timeframe was added as needed (e.g., ‘right now’ was added to Question 1; see **Table 2**). Similarly, few questions complied with the principles of prompting an open-minded response (e.g., ‘in an ideal world’) and fostering a collectivist perspective (e.g., ‘you or those you care for’). Questions that did not align with these principles were not excluded as the population-based study aimed to capture actual needs and experiences from the perspective of individual respondents.

From the 16 eligible questions, preferential voting by the investigator team resulted in a shortlist of 4 questions. In line with principles endorsed by workshop participants, team members were in favour of including prompts alongside the question, with the most popular prompts being: ‘Share as much detail as you would like to’ (*n* = 5, 71%) and ‘For example, you might like to consider your practical, emotional, psychological, financial, relational, and cultural needs’ (*n* = 5, 71%). **Table 4** summarises participants’ interpretations of and feedback on these questions and prompts in the interviews. Participants’ responses to the shortlisted questions are provided in **S5 Table** for comparison.

**Table 4:** Feedback from participants in the interviews regarding the four shortlisted questions for a population-wide study on the supportive care needs and experiences of people affected by cancer^a^.

Question^a^	Interpretation of the question	Feedback
**Option 1: Please tell us about your experiences since diagnosis and what would help you most right now?**	**Strengths** • Clearly addresses both experiences and needs. • The two-part structure encourages detailed responses. **Challenges** • The inclusion of two timeframes, ‘since diagnosis’ and ‘right now,’ can be confusing. Responses may be conflicting as experiences ‘since diagnosis’ may not align with support needs ‘right now’. Participants tended to describe their needs ‘since diagnosis’ rather than ‘right now’. • The term ‘right now’ lacks clarity, as it could refer to the past day or month. If referring to the past day, it may not capture the full scope of current needs. • Caregivers did not think ‘experiences since diagnosis’ was applicable to them because they had not received a cancer diagnosis themselves. Caregivers tended to answer this part of the question from the patient’s perspective rather than their own. • The timeframe ‘since diagnosis’ encompasses a long period of time, making it overwhelming for respondents to devise and communicate their response to this question.	Suggested changes to wording • Clarify what the timeframe ‘right now’ refers to. • Ask about one point in time or one phase of the cancer experience. Comparison with other questions • Option 1 and 2 are similar two-part questions. Wording of Option 1 is more complex than Option 2. • Options 1 and 2 are more inviting than Options 3 and 4.
**Option 2: How has cancer affected your life and what do you need right now?**	**Strengths** • Clearly addresses both experiences and needs. • The two-part structure encourages detailed responses. • ‘How has cancer affected your life?’ prompts emotional and reflective responses. ‘What do you need right now?’ encourages tangible and practical responses. • ‘How has cancer affected your life?’ encourages respondents to describe the ‘big picture’ issues that are impacting their life, such as changes to relationships and finances, rather than detailing a chronological story of events since diagnosis. • The wording is supportive because it focuses on the respondent as a whole person and emphasises that the research is aiming to help people affected by cancer. • The wording would be relevant to all stages of cancer, enabling respondents to answer this question at any timepoint. **Challenges** • The term ‘right now’ lacks clarity, as it could refer to the past day, week, or month. While it prompts respondents to consider their recent needs, some participants felt their needs were greater in the diagnosis or treatment stages of cancer and therefore answered retrospectively based on those needs. • If using a question that includes a short timeframe, there is a need to follow-up with people to ask the question again at a later stage (e.g., several months later) since their needs will have likely changed.	**Suggested changes to wording** • Clarify what the timeframe ‘right now’ refers to. **Comparison to other questions** • Options 1 and 2 are more inviting than Options 3 and 4. Participants felt it was easier to talk about experiences than needs. • Caregivers felt Option 2 was more relevant to them than Option 1 as cancer had affected their life, even though they were not personally diagnosed with cancer. • Option 2 was easier to read than Option 1. • Participants preferred the direct approach of Option 2 rather than the indirect approach of ‘Please tell us…’ in Options 1 and 3.
**Option 3: Please tell us how we can support you right now?**	**Strengths**• The wording is short, simple, and direct. • The wording would be relevant to all stages of cancer, enabling respondents to answer this question at any timepoint. • **Challenges**• Respondents may list their needs (e.g., money, transport) without elaborating further.• Although the term ‘right now’ prompts respondents to consider their recent needs, some participants felt their needs were greater in the diagnosis or treatment stages of cancer and therefore answered retrospectively based on those needs.• The term ‘right now’ emphasises daily needs. By using this term, longer term needs may be missed if they were not pressing in that moment.• Explicitly addresses needs but not experiences. It may be easier for respondents to describe how cancer is impacting their life rather than what they need. Asking about experiences may provide insight into needs or potential mechanisms underlying these needs, especially if respondents find it difficult to articulate their needs.• Some caregivers interpreted this question in relation to the patient’s needs.• Some participants felt that asking ‘How can we support you right now?’ suggests that Cancer Council Queensland will action their request for support in the near future.	**Suggested changes to wording** • Clarify what the timeframe ‘right now’ refers to. • Reword the question to ask people for their feedback, experience, or opinion. **Comparison to other questions** • Options 3 and 4 are similar but participants preferred the simplicity of Option 3. • Participants preferred Options 1 or 2 as these questions ask about experiences as well as needs.
**Option 4: What support do you need right now based on your experience?**	**Strengths** • Asking about needs ‘right now’ positions Cancer Council Queensland as an organisation that is able to deliver support in a timely manner. **Challenges** • The term ‘right now’ lacks clarity, as it could refer to the past week or month. • The phrase ‘based on your experience’ may not resonate with respondents who have recently been diagnosed with cancer or recently started caring for someone with cancer, as they may feel that they lack the experience needed to answer the question. • Explicitly addresses needs but not experiences. Peoples’ experiences may be implicit in their response to this question, but likely lacking in detail that may be important for contextualising their needs.	**Suggested changes to wording** • Reword ‘based on your experience’ to ‘based on your situation’ or ‘to ease your burden’. **Comparison to other questions** • Options 3 and 4 are similar but participants preferred the simplicity of Option 3. • Participants preferred Options 1 or 2 as these questions ask about experiences as well as needs.

All question options included the preamble: ‘At Cancer Council Queensland, we want to ensure that Queenslanders affected by cancer are provided with the support they need…’

Of the four shortlisted questions, interview participants preferred the two-part questions that explicitly asked about needs *and* experiences (i.e., Options 1 and 2) (see **Table 4**). Participants thought this question structure would generate richer information, particularly from respondents who find it difficult to articulate their needs. Of these two options, most participants (*n* = 13/14) endorsed Option 2 as it was easier to read and interpret, applicable to both cancer survivors and their caregivers, and relevant to any timepoint post-diagnosis. Participants recommended clarifying the timeframe that ‘right now’ referred to. For the question prompts, it was suggested to substitute complex words (i.e., replace ‘relational’ with ‘social’), remove words with similar meanings (i.e., remove ‘psychological’ and retain ‘emotional’), and broaden the scope (i.e., add ‘physical’ and ‘spiritual’). Option 2 was revised accordingly, with the wording of this question also changed to present tense to provide further clarification of the timeframe. These revisions resulted in the final question:

*Thinking about the past month, how is cancer affecting your life and what do you need? Share as much detail as you would like to. For example, you might like to consider your physical, emotional, practical, financial, social, cultural, and spiritual needs, or any other needs you might have.*

In the final six interviews, this question demonstrated capacity to elicit relevant, detailed data on the unmet supportive care needs and experiences of people affected by cancer (see **S5 Table**).

### Study invitation materials

Ten principles for designing study invitation materials were identified from participant feedback during the co-design workshops and validated during the interviews. These principles are presented in **Table 5** alongside an explanation and participant quotes. These principles were to: *communicate empathy and sensitivity; consider appropriate timing; convey credibility and legitimacy; facilitate reciprocal benefit; include a ‘human element’; increase accessibility and ease of participation; optimise readability; promote inclusivity; provide reassurance around privacy; motivate and incentivise participation;* and *support informed decisions*. An eleventh principle, *promote inclusivity*, emerged from the interview data only (see **Table 5**). **Table 5** also provides examples of how each principle was applied in this study to develop invitation materials for the proposed population-based survey. For example, following the co-design workshops, a flyer was created to accompany the letter, which included a personal quote from a cancer survivor emphasising the value of the research for people impacted by cancer.

**Table 5:** Principles for designing study invitation materials endorsed by participants during the co-design workshops and interviews for co-designing a population-wide study on the supportive care needs and experiences of people affected by cancer.

Principle	Explanation	Illustrative quotes from co-design workshops and interviews^a^	Application to population-wide study
**Communicate empathy and sensitivity**	Acknowledge that the recipient may be navigating a difficult period in their life. Avoid potentially dismissive, insensitive, or confusing statements that could contribute to distress.	• **P35:** “[It’s about] showing that you understand what [the recipients] are going through and meeting them where they are at, so it is more likely for them to want to engage. . . [Otherwise], they may feel it is out of touch with what they are going through”. **(Workshop C)** • **P20:** “You should put something in the letter like, ‘We understand that receiving a letter like this can induce stress or fear… We understand that you might have a great deal of fatigue at this time’”. **(Workshop H)** • **P26:** “I really like that [the flyer is] acknowledging that it’s a challenging time. . . because when you are newly diagnosed, you’ve got so much to deal with”. **(Interview)**	• Created a flyer to accompany the invitation letter. This flyer includes the statement, ‘We understand that this is a challenging time for you’. • Revised any statements in the invitation letter that participants identified as confusing or dismissive.
**Consider appropriate timing**	Avoid sending an invitation letter to an eligible participant too early within their cancer trajectory when it could induce stress or overwhelm. In addition, avoid sending an invitation letter after the individual has passed. Similarly, avoid sending reminder letters too soon or too late following the initial invitation.	• **P5:** “If I think back to when I was caring for Dad, I was already so short on time… It’d be meaningful to me but… it’d probably be too hard with everything else going on”. **(Workshop N)** • **P20:** “Some people on the Register may have passed away. It would be very upsetting for family members to receive a letter [addressed] to the person who has passed asking them to participate”. **(Workshop H)** • **P20:** “You don’t want to sort of go, ‘Oh, come on, get your act together’, or whatever, because… if you’re doing active cancer treatment, you feel terrible. . . So, you’ve got to give [recipients] a chance to get through their treatment for the month or for the week, have a good period, and then maybe pick it up”. **(Interview)** • **P17:** “A very short [reminder] window would be better because then you’ve got the accumulation effect of anything that [recipients] did take on board from the first letter… If you follow up quite quickly, it also gives a bit more clout to the importance [of the survey]”. **(Interview)**	• Recruitment methods following distribution of the invitation packs (including reminder letters) will be pilot tested to assess participation rates in the target population, stratified by time since diagnosis (i.e., 0–3 months, 3–6 months, 6–9 months, 9–12 months).
**Convey credibility and legitimacy**	Incorporate the branding (e.g., logo, colours, fonts) of the relevant affiliations to convey professionalism, and therefore credibility and legitimacy.	• **P34:** “Can you put your branding on there? If I saw [the invitation letter] coming from [Cancer Council Queensland], I would… probably be more likely to fill it out”. **(Workshop C)** • **P18:** “You need to have a letter with the Cancer Council Queensland logo and colours to give it some credibility”. **(Workshop I)** • **P47:** “[The flyer] might get my attention because it’s got Cancer Council Queensland right at the very top”. **(Interview)**	• Incorporated the branding of the relevant affiliations on the flyer and invitation letter, including logos and colours. • Included a brochure in the invitation pack that outlines information about the Queensland Cancer Register.
**Facilitate reciprocal benefit**	Use the opportunity of contacting people recently impacted by cancer to promote the supportive care services that are currently available to them. Explain how respondents may benefit from participating in the study, and how they will be advised on the research outcomes following their participation.	• **P23:** “With this opportunity to be able to contact thousands of people across Queensland, inform them of the services. Use this opportunity to give and take”. **(Workshop C)** • **P20:** “When a person sees something about doing a survey, they might ask themselves, ‘What’s in it for me?’ …I mean, it says, ‘Can you help us improve the support… available to Queenslanders affected by cancer?’ We could maybe reword that to, ‘Can you help us improve the support available to all Queenslanders affected by cancer, including you?’” **(Interview)** • **P42:** “[Include] a sentence that says how you will let people who contributed their lived experience know the outcomes of the study, so they feel included. If you need to go back and ask those same people another question, you are more likely to retain them. Instead of it being, ‘We got something from you, and you’ll never hear from us again’”. **(Interview)** • **P49:** “Given that this [flyer] is going to people that have just been diagnosed, I see huge benefit to actually helping them understand what [support] is available… sooner rather than later”. **(Interview)**	• Included information on the flyer about existing support services available at Cancer Council Queensland for people affected by cancer, including informal caregivers. • Included a statement on the invitation letter to convey how respondents may directly benefit from the research (i.e., ‘Some people also find that reflecting on and expressing their needs and experiences as part of a research study is a valuable opportunity for them’). • Added a statement on the flyer to direct recipients to the Cancer Council Queensland Research webpage, where outcomes from research projects are posted (i.e., ‘Find out more about our research here’).
**Include a ‘human element’**	Include images, stories, or quotes in recruitment materials to incorporate a ‘human element,’ which is warm and engaging to recipients. Avoid a clinical, formal, or factual tone in written text.	• **P20:** “The flyer could be from [the perspective of] someone who understands [the recipient’s] viewpoint, understands what it’s like to go through everything they are going through”. **(Workshop H)** • **P21:** “Add photos, positive photos. . . You’d feel calmer looking at it”. **(Workshop H)** • **P31:** “Centring the individual will make such a difference. End the letter with, ‘Your experience matters’ and ‘Your opinion is meaningful to us’. Everything you get, from scan results to the way your oncologist talks, is so clinical. . . Let’s make it accessible and humanise it”. **(Workshop D)** • **P16:** “If I get a letter that says, ‘Dear Mr [Surname]’, you’ve lost me… I am not so much engaged, and I think it’s much more serious than it is. If I get a letter that says, ‘Dear [First name]’, I’m much more relaxed in engaging with it”. **(Interview)** • **P31:** “‘Your input is meaningful’… ‘Input’ is a very formal, clinical word. I would simply change it to ‘Your story is meaningful’… ‘Input’ is just a very sort of computational word, like I’m inputting the data into the computer”. (**Interview**)	• Included colour, images, and a personal quote from a cancer survivor on the flyer. • Removed the clinical and formal tone from the invitation letter (e.g., recipients were addressed by their first name rather than their title and surname). • Included a heading within the invitation letter (i.e., ‘Your story is meaningful’). • Used second-person language in the invitation letter (e.g., ‘Invitation to participate in research study’ was changed to ‘We are inviting you to share your story with us’).
**Increase accessibility and ease of participation**	Ensure instructions for completing the survey are clear and explicit.	• **P31:** “For people who are older, they might have no idea what a QR code is and not know what to do with it, how to scan it. They might not have a smartphone. If that link is long and contrived, I’m not sure they will use it. It should be clear that there are three ways they could participate, [including] a paper survey”. **(Workshop D)** • **P16:** “I love all the options. You’re making it so easy. They can contribute a variety of ways. You’ve got a QR code. You’ve got a web link. You’ve got a phone number. You’ve got paper and pen. I mean, you can’t make it any easier really”. **(Interview)** • **P31:** “‘Prefer to enter another way? Visit the URL’. That’s great. The URL is short and simple. [It’s] easy to type into your browser”. **(Interview)**	• Included explicit instructions on the invitation letter and flyer for different ways that recipients can complete the survey (e.g., ‘To answer this question, please scan this QR code using a smartphone camera. Prefer to answer another way? Visit the website, call us, or use the paper form enclosed’).
**Promote inclusivity**	Use language that is inclusive and inviting of a diverse range of responses. Ensure that different groups are represented on the materials.	• **P11:** “The first thing that stood out to me was the picture of the people down the bottom… A lot of young people are affected by a cancer as well, and I feel like that’s just pointed at older generations, which potentially could make people think that they don’t fit into that category of needing help. Yeah, maybe [include] a little bit more representation there of different ages and maybe a bit of diversity”. **(Interview)** • **P26:** “I like the fact that [the flyer is] talking about the patient and their loved ones. So, it’s not solely directed at the patient, which is good because you obviously recognise that it affects other people in the family”. **(Interview)** • **P31:** “‘You can write as little or as much as you like’. That’s excellent. That’s very welcoming and inclusive”. **(Interview)**	• Included an image on the flyer that represents people of different ages. • Included a statement on the invitation letter that encourages a range of different responses (i.e., ‘You can write as little or as much as you like’). • Used language in the invitation letter and flyer that was inclusive of people with a personal diagnosis of cancer and those who provide support (e.g., ‘Your involvement in this study can help CCQ develop and improve the support programs available to cancer survivors and their families across Queensland’).
**Provide reassurance around privacy**	Provide reassurance for the privacy of personal information since recipients will be identified and invited from a population-based register. Explain how data will be used and shared in the research.	• **P1:** “[It says], ‘We found your information through [the Queensland Cancer Register]’, but if you don’t even know you’re on this list, it could be a bit confronting”. **(Workshop O)** • **P29:** “One of the things I think about when I’m answering is confidentiality. If you want me to get specific about anything, I get pretty cagey online until I know it’s going to be dealt with confidentially”. **(Workshop E)** • **P26:** “I think it’s good that you let [recipients] know that [their data] is confidential. It’s not that random people have been given their information to send letters out… If I’d got something like this, I’d be like, ‘Well, how do you know [my details]?’. . .So, it’s good to get information [about the Queensland Cancer Register] because some people probably don’t know what it is”. **(Interview)**	• Added information about data confidentiality to the invitation letter (e.g., ‘Your name or any information about you will not be given to the research team at Cancer Council Queensland without your permission’). • Included a brochure in the invitation pack that outlines information about the Queensland Cancer Register.
**Motivate and incentivise participation**	Motivate recipients to participate in the survey by including a direct call to action, and articulating how their response would contribute to better outcomes for others. Emphasise the novelty of the study to generate interest and appeal.	• **P15:** “List key things that you are going to deliver on. That would make me think, ‘My input on this is worthwhile… That would have really helped me’. I would feel compelled to contribute to this so I can help others. Right now, I read that, and I think, ‘That means nothing to me. Cancer support services… I don’t know what they are, didn’t use them, don’t care. . . What’s in this for me? I don’t know”. **(Workshop J)** • **P11:** “It says, ‘Your response will help provide the emotional and practical support that people need the most’. Yeah, that made me go, ‘Yep, I would [participate]’. [There’s] information about how you’re going to use [the data]”. **(Interview)** • **P31**: “‘We are inviting you to share your story with us as part of our new research study’. . .I think the appeal of the word ‘new’ is that you might think, ‘Oh, great. This is new. This is innovative. This isn’t stale. This isn’t something that has been in the pipeline for years. That’s exciting. I wonder what it is’ …I might be more likely to participate”. **(Interview)**	• Included statements in the flyer and invitation letter to convey the value of respondents’ contributions to the research (e.g., ‘Help us improve the support available to Queenslanders affected by cancer,’ ‘Your response will help Cancer Council Queensland provide the emotional and practical support that people need most’). • Included the word ‘new’ when describing the research study in the invitation letter (e.g., ‘We are inviting you to share your story with us as part of a new research study by Cancer Council Queensland’) and included the version number and date of the invitation letter in a footnote.
**Support informed decisions**	Outline key information to inform decisions about participation (e.g., research aims and outcomes, time involved, funding). Explain that the research is voluntary, and if applicable, does not involve donating or participating in a clinical trial, as recipients may affiliate cancer research with these activities.	• **P23:** “Do I have to donate? …I hate to be cynical, but that’s how most people will look at emails, text messages, or letters… So, defining that it is not that at all–‘We want to help you have better access to more services”. **(Workshop G)** • **P42:** “Who is funding it? …That is important to me. This letter doesn’t answer those questions. That means as a time-poor mum, I would have to seek answers to those questions”. **(Workshop A)** • **P31:** “I think emphasising that you only have one question is effective because immediately you have an idea of how much time you need to donate to this [survey]”. **(Interview)** • **P46:** “I think to me it reads pretty clearly and kind of outlines what the study is, that it won’t take that long, that there’s a bunch of ways to do it… Yeah, [they are] the main points”. **(Interview)**	• Outlined details about participation in the invitation letter (e.g., ‘Taking part in this study involves answering **just one question** about your experiences and needs in the past month. This will take around five minutes of your time’ and ‘Taking part in the study is entirely voluntary’). • Specified in the invitation letter that the study has received ethical approval and is being supported by Cancer Council Queensland.
**Optimise readability**	Use clear and concise language, and consistent and logical formatting. Organise and emphasise key information using subheadings, bullet points, colour, and bolded, underlined, or italicised text. Ensure that the font size is sufficiently large.	• **P23:** “Consider bullet points rather than paragraphs… People [would be] speed reading or skimming it. . . People don’t sit down and read a letter”. **(Workshop G)** • **P36:** “There is a lot to read. When you are going through a horrible treatment, the less to read, [the better]. A shorter version would entice people to fill [the survey] out”. **(Workshop C)** • **P9:** “It sort of looks like there’s four things I have to do, rather than four different ways [to participate]… Dot points would be fine… because numbers look like I have to do four steps”. **(Interview)** • **P15:** “I didn’t feel engaged [when I read the invitation letter in the workshop]. This [version] feels much, much more engaging… The colour, the layout, the way it’s just simple to kind of digest. . . Before I was like, ‘No, too hard, throw it away, like too many words’”. **(Interview)** • **P20:** “The writing is very small. Under that [QR] code, even with my glasses on… I can’t even read that”. **(Interview)**	• Simplified the language used in the invitation letter and flyer (e.g., ‘take part’ rather than ‘participate’) and used acronyms where relevant. • Applied text formatting and colour to the invitation letter and flyer to emphasise key information and increased the overall font size used. • Used a dot point list in the invitation letter to outline different ways that recipients can participate in the survey.

Participants have been designated a code to maintain their anonymity.

The revised invitation materials presented to participants in the 20 interviews were highly accepted, with 19 (95%) participants indicating they would likely respond to the qualitative survey question. The one participant who indicated they would not complete the survey was reluctant to scan a quick response (QR) code. Therefore, instructions on alternative methods for completing the survey were emphasised on the invitation materials in the final revision stage, alongside several other minor changes, such as increasing the font size and simplifying the language used.

## Discussion

This qualitative study used co-design methods to develop and test study materials for capturing the supportive care needs and experiences of cancer survivors and their caregivers in a future population-based survey. Principles for designing qualitative data collection tools and study invitation materials were identified in workshops with community members and validated in interviews. These principles can be used more broadly by health and survivorship researchers to design study materials that align with community preferences.

To date, studies examining strategies to optimise participation in cancer research have focused primarily on methods of advertisement (e.g., social media, text messages), incentives, eligibility criteria, and outcome measures [28, 29]. However, few studies have investigated community preferences for invitation materials. A conceptual model developed by Chhatre and colleagues [30] describes strategies for recruiting cancer patients into clinical trials using a patient-centred approach. Recommended strategies mapped onto four concepts, including trust, communication, expectations, and attitudes, and ranged from protecting patient health information to emphasising the altruistic value of research involvement [30]. These findings align with principles endorsed by community members in the current study, which supports the applicability of these principles across clinical and observational research settings.

To the authors’ knowledge, few studies have reported on the development and testing of qualitative surveys for health research, despite the importance of question wording in survey-based studies [31]. Principles identified in the current study both validate and expand on those previously identified in best practice guidelines for qualitative research [31–33]. For example, similar to consumers in this study, Braun and colleagues [31] recommend that survey questions are short and unambiguous, void of assumptions, and include examples to guide the scope of responses. Similar to other widely used surveys [10–12], a timeframe was also added to the question (i.e., the past month) to minimise participant burden when responding, enable repeated data collection over time, and allow comparability of responses by time since diagnosis. The current study also identified additional principles for designing a qualitative survey. For example, leveraging the expertise of respondents by asking them to share their experiences, rather than just their needs, enables researchers to identify trends and gaps in care at the population-level. Considering that the principles in this study were endorsed by a diverse sample of cancer survivors and caregivers, the principles identified may also be applicable to studies investigating the supportive care needs and experiences of people affected by other health conditions.

The single, open-ended survey question designed in the current study was developed to collect rich, detailed information on the supportive care needs and experiences of people affected by cancer for the purpose of informing service delivery. Current literature on supportive care needs is largely based on data derived from quantitative measures. These measures are confined to specific supportive care domains, with needs in the cognitive, spiritual, sexual, or financial domains often overlooked or excluded from shorter versions of tools [14, 34]. In contrast, the question developed in this study asks about supportive care needs in the context of participants’ experiences (i.e., how cancer is affecting their life). Based on participant feedback in the workshops, the question includes a prompt that lists various domains of potential need. When this prompt was tested in interviews, its inclusion did not appear to constrain or direct participant responses. Therefore, the question provides an opportunity for respondents to share their experiences and to define what is important to them. The final question demonstrated applicability and acceptability among a diverse sample of cancer survivors and caregivers. Thus, the question presents an acceptable and potentially effective method for assessing the supportive care needs and experiences of people affected by cancer.

In the current study, there were some divergent views among community members regarding how to design a qualitative survey question. For example, some participants suggested asking about supportive care needs in an open-minded manner, using phrases such as ‘in an *ideal* world’ and ‘what support do you *wish* you had’. However, this principle conflicted with other participants’ suggestions to define the scope of the question by providing realistic examples of what supportive care services may be provided, to manage expectations of the research outcomes. Similarly, some participants noted that using terms like ‘ideal’ could be insensitive as they may imply a sense of choice or control in a person’s cancer trajectory. Therefore, the principle of asking about needs in an open-minded manner was not applied in the current study but may be suitable to other populations or contexts depending on the research aims and scope.

### Strengths and limitations

This study used an iterative qualitative design, facilitating active and repeated engagement of community members in developing and testing study materials. Including a subset of participants in both phases of consumer consultation enabled the principles identified in workshops to be validated through member checking in interviews [24]. The use of two different consultation methods was another key strength; the workshops fostered collaborative group discussion which supported idea generation, while the interviews provided a platform for in-depth exploration of individual perspectives and experiences [24]. Finally, the study included a diverse sample, involving people who were born overseas, used English as a second language, identified as Aboriginal and/or Torres Strait Islander, and lived in a rural area.

The main limitation of this study is the potential for self-selection bias. The consumer consultations relied on individuals agreeing to participate in a research study and being able to speak English. Given that an estimated 900,000 people in Australia have low proficiency in spoken English [35], and that CALD groups experience poorer health outcomes compared to the general population [36], future research should work with non-English speaking people to design study materials that facilitate their participation in cancer research [37]. Additionally, the current study did not collect data on other characteristics, such as participants’ sexuality, education, and income. It is therefore unknown whether the findings represent diversity within these groups.

## Conclusions

Through active and repeated consultation with community members, this study identified principles for designing qualitative data collection tools and study invitation materials for use in cancer survivorship research. These principles were used to design and test an open-ended survey question and study invitation materials for use in a population-based study of the supportive care needs and experiences of cancer survivors and their caregivers. These principles can also be used by other researchers to optimise community participation in their qualitative research and to inform support service providers about the needs and experiences of consumers.

## Supporting information

S1 Table Completed Consolidated Criteria for Reporting Qualitative Research (COREQ) checklist. (DOCX)

S2 Table Overview of protocol for co-design workshops. (DOCX)

S3 Table Expertise of the co-investigator research team who voted on the questions generated from the co-design workshops for inclusion in the interviews. (DOCX)

S4 Table Overview of protocol for interviews. (DOCX)

S5 Table Responses to the shortlisted and final question in the interviews. (DOCX)

## References

[1] Coleman MP, Forman D, Bryant H, Butler J, Rachet B, Maringe C, et al. Cancer survival in Australia, Canada, Denmark, Norway, Sweden, and the UK, 1995–2007 (the International Cancer Benchmarking Partnership): An analysis of population-based cancer registry data. Lancet. 2011;377:127–38. doi: 10.1016/S0140-6736(10)62231-3 21183212 PMC3018568

[2] Sung H, Ferlay J, Siegel RL, Laversanne M, Soerjomataram I, Jemal A, et al. Global Cancer Statistics 2020: GLOBOCAN estimates of incidence and mortality worldwide for 36 cancers in 185 countries. CA Cancer J Clin. 2021;71:209–49. doi: 10.3322/caac.21660 33538338

[3] Wu H-S, Harden JK. Symptom burden and quality of life in survivorship: A review of the literature. Cancer Nurs. 2015;38:E29–54. doi: 10.1097/NCC.0000000000000135 24831042

[4] Harrison R, Raman M, Walpola RL, Chauhan A, Sansom-Daly UM. Preparing for partnerships in cancer care: An explorative analysis of the role of family-based caregivers. BMC Health Serv Res. 2021;21:620. doi: 10.1186/s12913-021-06611-0 34187469 PMC8240189

[5] Teixeira RJ, Remondes-Costa S, Graça Pereira M, Brandão T. The impact of informal cancer caregiving: A literature review on psychophysiological studies. Eur J Cancer Care (Engl). 2019;28:e13042. doi: 10.1111/ecc.13042 30990936

[6] World Health Organization. Health employment and economic growth: An evidence base. World Health Organization; 2017. Available from: https://iris.who.int/handle/10665/326411

[7] Park M, Giap T-T-T, Lee M, Jeong H, Jeong M, Go Y. Patient- and family-centered care interventions for improving the quality of health care: A review of systematic reviews. Int J Nurs Stud. 2018;87:69–83. doi: 10.1016/j.ijnurstu.2018.07.006 30056169

[8] Tian L, Cao X, Feng X. Evaluation of psychometric properties of needs assessment tools in cancer patients: A systematic literature review. PLOS ONE. 2019;14:e0210242. doi: 10.1371/journal.pone.0210242 30620770 PMC6324833

[9] Prue G, Santin O, Porter S. Assessing the needs of informal caregivers to cancer survivors: A review of the instruments. Psychooncology. 2015;24:121–9. doi: 10.1002/pon.3609 24930811

[10] Boyes A, Girgis A, Lecathelinais C. Brief assessment of adult cancer patients’ perceived needs: Development and validation of the 34-item Supportive Care Needs Survey (SCNS-SF34). J Eval Clin Pract. 2009;15:602–6. doi: 10.1111/j.1365-2753.2008.01057.x 19522727

[11] Hodgkinson K, Butow P, Hunt GE, Pendlebury S, Hobbs KM, Lo SK, et al. The development and evaluation of a measure to assess cancer survivors’ unmet supportive care needs: The CaSUN (Cancer Survivors’ Unmet Needs measure). Psychooncology. 2007;16:796–804. doi: 10.1002/pon.1137 17177268

[12] Shin DW, Park J-H, Shim E-J, Park J-H, Choi J-Y, Kim SG, et al. The development of a comprehensive needs assessment tool for cancer-caregivers in patient-caregiver dyads. Psychooncology. 2011;20:1342–52. doi: 10.1002/pon.1857 22102594

[13] Girgis A, Lambert S, Lecathelinais C. The supportive care needs survey for partners and caregivers of cancer survivors: Development and psychometric evaluation. Psychooncology. 2011;20:387–93. doi: 10.1002/pon.1740 20878835

[14] Hart NH, Crawford-Williams F, Crichton M, Yee J, Smith TJ, Koczwara B, et al. Unmet supportive care needs of people with advanced cancer and their caregivers: A systematic scoping review. Crit Rev Oncol Hematol. 2022;176:103728. doi: 10.1016/j.critrevonc.2022.103728 35662585

[15] Slattery P, Saeri AK, Bragge P. Research co-design in health: A rapid overview of reviews. Health Res Policy Syst. 2020;18:17. doi: 10.1186/s12961-020-0528-9 32046728 PMC7014755

[16] Domecq JP, Prutsky G, Elraiyah T, Wang Z, Nabhan M, Shippee N, et al. Patient engagement in research: A systematic review. BMC Health Serv Res. 2014;14:89. doi: 10.1186/1472-6963-14-89 24568690 PMC3938901

[17] Willis GB, Artino AR. What do our respondents think we’re asking? Using cognitive interviewing to improve medical education surveys. J Grad Med Educ. 2013;5:353–6. doi: 10.4300/JGME-D-13-00154.1 24404294 PMC3771159

[18] Tong A, Sainsbury P, Craig J. Consolidated criteria for reporting qualitative research (COREQ): A 32-item checklist for interviews and focus groups. Int J Qual Health Care J. 2007;19:349–57. doi: 10.1093/intqhc/mzm042 17872937

[19] Australian Institute of Health and Welfare. Rural and remote health. Australian Government; 2023. Available from: https://www.aihw.gov.au/reports/rural-remote-australians/rural-and-remote-health

[20] Ambroggi M, Biasini C, Del Giovane C, Fornari F, Cavanna L. Distance as a barrier to cancer diagnosis and treatment: Review of the literature. Oncologist. 2015;20:1378–85. doi: 10.1634/theoncologist.2015-0110 26512045 PMC4679078

[21] Health Consumer Queensland. Health Consumers Queensland 2024. Available from: https://www.hcq.org.au/.

[22] Harris PA, Taylor R, Thielke R, Payne J, Gonzalez N, Conde JG. Research electronic data capture (REDCap)—A metadata-driven methodology and workflow process for providing translational research informatics support. J Biomed Inform. 2009;42:377–81. doi: 10.1016/j.jbi.2008.08.010 18929686 PMC2700030

[23] Harris PA, Taylor R, Minor BL, Elliott V, Fernandez M, O’Neal L, et al. The REDCap consortium: Building an international community of software platform partners. J Biomed Inform. 2019;95:103208. doi: 10.1016/j.jbi.2019.103208 31078660 PMC7254481

[24] Liamputtong P. Qualitative Research Methods. Melbourne: Oxford University Press Australia and New Zealand; 2020.

[25] McMillan SS, King M, Tully MP. How to use the nominal group and Delphi techniques. Int J Clin Pharm. 2016;38:655–62. doi: 10.1007/s11096-016-0257-x 26846316 PMC4909789

[26] Willis G. Cognitive interviewing in survey design: State of the science and future directions. In: Vannette DL, Krosnick JA, editors. Palgrave Handbook of Survey Research. Cham: Palgrave Macmillan; 2018, p. 103–7. doi: 10.1007/978-3-319-54395-6_14

[27] Elo S, Kyngäs H. The qualitative content analysis process. J Adv Nurs. 2008;62:107–15. doi: 10.1111/j.1365-2648.2007.04569.x 18352969

[28] Wang RR, Schweitzer JB, Hernandez S, Molina SC, Keegan TH. Strategies for recruitment and retention of adolescent and young adult cancer patients in research studies. J Clin Transl Sci. 2023:1–27. doi: 10.1017/cts.2023.669 38028342 PMC10663769

[29] Liu J, Gutierrez E, Tiwari A, Padam S, Li D, Dale W, et al. Strategies to improve participation of older adults in cancer research. J Clin Med. 2020;9:1571. doi: 10.3390/jcm9051571 32455877 PMC7291007

[30] Chhatre S, Jefferson A, Cook R, Meeker CR, Kim JH, Hartz KM, et al. Patient-centered recruitment and retention for a randomized controlled study. Trials. 2018;19:205. doi: 10.1186/s13063-018-2578-7 29587805 PMC5870194

[31] Braun V, Clarke V, Boulton E, Davey L, McEvoy C. The online survey as a qualitative research tool. Int J Soc Res Methodol. 2021;24:641–54. doi: 10.1080/13645579.2020.1805550

[32] Braun V. Successful qualitative research: A practical guide for beginners. London: SAGE Publications Ltd; 2013.

[33] Patton M. Qualitative research & evaluation methods. 4th ed. California: SAGE Publications Ltd; 2015.

[34] Lisy K, Langdon L, Piper A, Jefford M. Identifying the most prevalent unmet needs of cancer survivors in Australia: A systematic review. Asia Pac J Clin Oncol. 2019;15:e68–78. doi: 10.1111/ajco.13176 31215167

[35] Australian Bureau of Statistics. Proficiency in spoken English (ENGLP) 2022. https://www.abs.gov.au/articles/cultural-diversity-australia (accessed March 12, 2024).

[36] Butow PN, Aldridge L, Bell ML, Sze M, Eisenbruch M, Jefford M, et al. Inferior health-related quality of life and psychological well-being in immigrant cancer survivors: A population-based study. Eur J Cancer. 2013;49:1948–56. doi: 10.1016/j.ejca.2013.01.011 23465493

[37] Charles RHC, Sosa E, Patel M, Ertunmwunsee L. Health disparities in recruitment and enrollment in research. Thorac Surg Clin. 2022;32:75–82. doi: 10.1016/j.thorsurg.2021.09.012 34801198 PMC8611804

