# Supplementary material for: From many voices, one question: Community co-design of a population-based qualitative cancer research study
Source: PLoS One. 2024 Aug 26;19(8):e0309361. doi: 10.1371/journal.pone.0309361 (PMC11346942; doi:10.1371/journal.pone.0309361)
Supplement: S2 Table — (DOCX) [file pone.0309361.s002.docx]

# **S2 Table.** Overview of protocol for co-design workshops.

| Protocol | |
| --- | --- |
| Equipment | |
| *In-person workshops:*   - Food and drink catering - Name tags - Pens, paper, and sticky notes - Printed demographic surveys - Printed and laminated prompt cards | - Printed recruitment letters - Laptop and screen with presentation slides - Dictaphone   ***Online workshops:***   - Microsoft Teams |
| Set up/preparation | |
| - Ask participants to write their name on a name tag [in-person only] - Ask participants to complete the demographic survey [send link to online participants] | |
| Introduction | |
| Welcome participants | |
| Introduce the research team | |
| Provide an overview of the workshop | |
| - Administrative tasks [start audio-recording with permission from participants] - Guidelines and expectations | |
| Allow participants to introduce themselves | |
| Provide overview of the vision and mission of Cancer Council Queensland (CCQ) | |
| Introduce UNIQUE | |
| - Background and aims - Recruitment - Data collection | |
| Outline purpose of the workshop | |
| Activity 1: Survey question | |
| Introduce activity | |
| Explain criteria for the survey question | |
| - Provide examples on presentation slides - Provide preamble to question on presentation slides  – i.e., *We want to ensure that all Queenslanders affected by cancer are provided with the support they need to live well. As a person affected by cancer in Queensland…* | |
| Ask participants to individually write a list of survey question ideas (10 minutes) | |
| - Provide pen and paper [in-person only] - Provide prompt cards [type prompts into chat for online participants]  – e.g., *What do you wish you were asked at the time of diagnosis? Imagine you or a loved one are facing a cancer diagnosis. What question would you want to answer to express your needs and experiences?* | |
| Invite participants to share their survey question ideas with the group following a ‘round-robin’ process | |
| - Type questions verbatim onto the presentation slides | |
| Facilitate a group discussion using the survey question ideas as prompts | |
| – e.g., *What was your thinking behind this question?* | |
| Ask participants to privately vote for their two most preferred survey question ideas | |
| - Provide pen and sticky notes [in-person only] - Create poll in Microsoft Teams [online only] | |
| 5-minute break | |
| - Tally the votes - Type the 2-3 highest ranked survey question ideas onto the presentation slides | |
| Facilitate a group discussion using the survey question ideas as prompts | |
| – e.g., *Should we include additional instructions or prompts with this question?* | |
| Activity 2: Recruitment material | |
| Introduce activity | |
| Provide overview of the recruitment methods | |
| Ask participants to read a draft version of the invitation letter   - Display a digital version of the letter on the presentation slides - Provide paper version of the letter [in-person only] | |
| Facilitate a group discussion using the invitation letter as a prompt | |
| – e.g., *What do you think about the wording of this letter?* | |
| Ask participants for their opinion on the timing for sending this letter | |
| – e.g., *In your opinion, how early can we invite people to take part in the survey following their diagnosis?* | |
| Conclusion | |
| Invite participants to share additional thoughts or feedback | |
| Invite participants to register for the second phase of the study (interviews) | |
| - Provide study flyer [email digital copy to online participants] | |
| Thank participants for their contributions | |
