# Supplementary material for: From many voices, one question: Community co-design of a population-based qualitative cancer research study
Source: PLoS One. 2024 Aug 26;19(8):e0309361. doi: 10.1371/journal.pone.0309361 (PMC11346942; doi:10.1371/journal.pone.0309361)
Supplement: S3 Table — (DOCX) [file pone.0309361.s003.docx]

# **S3 Table.** Expertise of the co-investigator research team who voted on the questions generated from the co-design workshops for inclusion in the interviews.

| Position | Organisation type | Area of expertise | Years working in oncology |  | Focus of work |
| --- | --- | --- | --- | --- | --- |
| Professor | University | Digital health | 16 |  | AI-assisted technology, childhood cancer |
| Associate Professor | Medical research institute | Supportive care and behavioural science | 20 |  | Supportive care self-reported outcome and intervention studies, patients and caregivers |
| Associate Professor | Not-for-profit | Behavioural science and survivorship | 8 |  | Health psychology, population-level behaviours and interventions |
| Postdoctoral researcher | Not-for-profit | Survivorship | 5 |  | Rural cancer survivors and caregivers, health behaviours, support seeking |
| Director of Medical Oncology | Rural-based hospital | Medical oncology | 10 |  | Upper gastrointestinal, pancreato-biliary, central nervous system, melanoma, breast, lung, colorectal, prostate cancers |
| Deputy Director, Division of Cancer Services | Metropolitan-based hospital | Medical oncology | 14 |  | Head and neck, genitourinary cancers |
| Executive | Not-for-profit | Supportive care and service delivery | 11 |  | State-wide operations and service delivery |
| Manager | Population-based registry | Population clinical data | 19 |  | Data management and quality |

^a^ Of the 8 research team members listed, a total of 7 responded to the online anonymous survey.
