# Supplementary material for: From many voices, one question: Community co-design of a population-based qualitative cancer research study
Source: PLoS One. 2024 Aug 26;19(8):e0309361. doi: 10.1371/journal.pone.0309361 (PMC11346942; doi:10.1371/journal.pone.0309361)
Supplement: S4 Table — (DOCX) [file pone.0309361.s004.docx]

# **S4 Table.** Overview of protocol for interviews.

| Protocol |
| --- |
| Equipment |
| - Laptop and screen with presentation slides - Microsoft Teams |
| Introduction |
| Welcome participant |
| Introduce self |
| Provide an overview of the interview |
| - Administrative tasks [start audio-recording with permission from participant] |
| Provide overview of the vision and mission of Cancer Council Queensland (CCQ) |
| Briefly introduce UNIQUE |
| - Background and aims - Recruitment - Data collection |
| Discuss Phase 1 of the current study (codesign workshops) |
| - Participants - Outcomes |
| Outline purpose of the interview |
| Activity 1: Revised recruitment material |
| Introduce activity |
| Ask participant to read the study flyer |
| - Display digital version of the study flyer on the presentation slides |
| Invite participant to share their feedback on the study flyer |
| – *e.g., What are your thoughts on the flyer? Would you change anything?* |
| Ask participant to read the invitation letter |
| - Display digital version of the invitation letter on the presentation slides |
| Invite participant to share their feedback on the invitation letter |
| – *e.g., What are your thoughts on the invitation letter? Would you change anything?* |
| Activity 2: Survey question |
| Introduce activity |
| Explain process for generating and shortlisting the survey questions |
| Survey question 1: Ask participant to read and respond to the survey question using the chat function (5 minutes)   - Display the survey question on the presentation slides |
| Introduce the ‘think aloud’ method |
| - Demonstrate an example |
| Survey question 1: Ask participant to talk through their interpretation of the survey question using the ‘think aloud’ method |
| *– e.g., Please read the question out loud and tell me what you’re thinking.* |
| Ask participant to read other alternative questions and indicate their preference for wording   - Display the survey questions on the presentation slides |
| Conclusion |
| Collect demographic data from participant (using structured verbal questions) |
| Invite participant to share additional thoughts or feedback |
| Thank participant for their contributions |
